# Higher temperatures and being an ethnic minority reduce mosquito net use in Lao PDR: an analysis of Lao PDR’s Multiple Indicator Cluster Survey and Earth observation satellite data

**DOI:** 10.1186/s41182-024-00669-2

**Published:** 2024-12-24

**Authors:** Wills Peter Otieno, Eunice Abena Kwatemaa Ankapong, Kanae Nomura, Emilie Louise Akiko Matsumoto-Takahashi

**Affiliations:** 1 Graduate School of Public Health, St. Luke’s International University, CCA 5th Floor, 3-6-2 Tsukiji, Chuo-Ku, Tokyo, 104-0045 Japan; 2 Department of Tropical Medicine and Malaria, Research Institute, National Center for Global Health and Medicine (NCGM), Tokyo, Japan

**Keywords:** Lao PDR, Mosquito net, Malaria, Multiple Indicator Cluster Survey, Earth observation satellite data

## Abstract

**Background:**

Malaria remains the leading cause of under-five morbidity and mortality in low- and middle-income countries. Sleeping under mosquito nets, especially insecticide-treated nets (ITNs), is one of the best ways to prevent malaria as they form a physical and chemical barrier against mosquitoes. Therefore, the present study aimed to assess not only mosquito net use, but also how environmental factors, specifically land surface temperature, contribute to malaria prevention among households with children under 5 years of age in Lao PDR.

**Methods:**

The most recent Multiple Indicator Cluster Survey datasets of Lao PDR 2017 and the Japan Aerospace Exploration Agency (JAXA) Public Health Monitoring and Analysis Platform (JPMAP) were used. Data from 51,948 households were used in the analysis. A bivariate logistic regression analysis was followed by a multivariate logistic regression analysis to determine the factors influencing mosquito net use with children under five.

**Results:**

In this study, 77.8% of all households with children under 5 years of age slept under mosquito nets. Of these, 80.5% were ITNs (Olyset, Permanent, and other brands). Multivariate logistic regression analysis revealed that mosquito net use was significantly associated with the land surface temperature, ethno-linguistic group (Lao-Tai, Mon-Khmer, Hmong-Mien, Chinese-Tibetan, and other), education level of the household head, and wealth index quintile.

**Conclusions:**

The analysis of the present study suggested measures to intensify the use of mosquito nets with an emphasis on ethnic minorities living in hot areas to bring Lao PDR closer to the day that mosquito-borne infections such as malaria can be eliminated.

**Supplementary Information:**

The online version contains supplementary material available at 10.1186/s41182-024-00669-2.

## Introduction

Malaria remains a significant public health challenge, with global estimates that indicated 249 million clinical cases annually in the year 2022, well above the estimated number of cases before the COVID-19 pandemic, and an increase of five million over 2021 [1]. The spread of malaria is closely linked to the receptivity and vulnerability of a specific area. According to the World Health Organization (WHO), receptivity refers to the extent to which the ecosystem in a particular location at a specific time supports the transmission of *Plasmodium* species from one person to another via mosquito vectors. Weather parameters affect the life cycle of vector mosquitoes and part of the life cycle of parasites/viruses inside vector and biting habits of vector mosquitoes [2].

To address this, mosquito nets are an important tool in the prevention of mosquito-borne infectious diseases, particularly malaria [3]. Mosquito nets, including insecticide-treated nets (ITNs), act as physical barriers that block access by vector mosquitoes and provide personal protection against malaria for individuals using the nets, especially while sleeping. The addition of pyrethroid insecticides enhances this protection by creating a chemical barrier, reducing human–vector contact, and increasing the nets’ efficacy. Insecticide-treated nets (ITNs) play a crucial role in malaria prevention by disrupting multiple aspects of mosquito behavior and physiology. ITNs not only reduce the duration of mosquito biting activity, but also limit the blood-meal size, directly impacting mosquito fecundity. Furthermore, ITNs exhibit a lethal effect, killing approximately 15% of mosquitoes within 24 h post-feeding [1]). This community-wide impact extends protection to all individuals in the community, even those not directly using the nets [4].

ITNs have proven to be a cornerstone in the global fight against various disease-transmitting vectors beyond malaria, such as leishmaniasis, Japanese encephalitis, lymphatic filariasis, and Chagas disease [4]. In addition to malaria, other mosquito-borne infections, such as dengue, Zika, Chikungunya, Japanese encephalitis, and filariasis, are prevalent in Lao PDR, though malaria remains the leading cause of morbidity. Most research evidence regarding the effectiveness of insecticide-treated nets (ITNs) focuses on malaria prevention, with limited impact on *Aedes*-borne diseases due to the differing biting habits of *Aedes* mosquitoes. *Aedes* species, responsible for transmitting diseases such as dengue and Zika, are day-biting, which reduces the likelihood of ITNs providing consistent protection against these diseases. However, ITNs are likely to be effective against multiple vectors and vector-borne diseases (VBDs), as a substantial proportion of transmission occurs indoors, particularly during nighttime when nets are in use.

The cost-effectiveness of exceptionally long-lasting insecticidal nets (LLINs) has gained attention for their impact on reducing disease burden and mortality rates. Recent analyses comparing the cost of implementing ITN programs have highlighted the cost advantages of LLINs over conventionally treated nets, with significantly lower costs per death averted and per disability-adjusted life year averted [4]. LLINs offer substantial cost savings compared to indoor residual spraying programs, making them an integral component of national anti-malaria programs [5, 6].

Lao People’s Democratic Republic (Lao PDR) continues to face significant challenges regarding mosquito-borne infectious diseases, notably malaria and dengue fever [3]. These diseases pose substantial public health burdens in the country, particularly in rural and remote areas where healthcare infrastructure may be limited. Malaria remains a major health concern in Lao PDR, especially in the south where transmission rates are higher [7]. *Plasmodium falciparum* and *P. vivax* are the predominant malaria parasites in the country [8]. Dengue fever is endemic to Lao PDR and outbreaks occur regularly, particularly during the rainy season when mosquito populations increase. The country experiences all four serotypes of the dengue virus, increasing the risk of severe dengue and complications [9–11]. Efforts to control these diseases include the distribution of LLINs, indoor residual spraying, and access to effective diagnosis and treatment [12]. Vector control measures, including the use of LLINs, are crucial in reducing the transmission.

Several studies have shown the effectiveness of LLINs in Lao PDR. A study conducted by WHO and the Lao Ministry of Health found a significant reduction in malaria incidence among individuals who consistently used LLINs compared to those who did not [13]. Additionally, the Lao National Malaria Control Programme integrates LLIN distribution as a key strategy in its efforts to eliminate malaria [14].

Despite the effectiveness of LLINs, challenges remain in ensuring universal access and sustained usage, especially among children under 5 years of age who are at high risk of infection [1]. Moreover, climate change is expected to alter the global footprint of many infectious diseases, particularly vector-borne diseases such as malaria and dengue [2, 3]. Therefore, this study aimed to evaluate mosquito net use and identify influencing factors in households with children under the age of five who are a more vulnerable group for infections in Lao PDR, especially focusing on a weather factor, land surface temperature (LST). This study also examines the association between land surface temperature (LST) and mosquito net use to understand how environmental factors contribute to malaria prevention. This will help people in Lao PDR to promote behavioral changes related to mosquito net utilization.

## Methodology

### Study design and data collection

This study used secondary cross-sectional data from the Multiple Indicator Cluster Survey (MICS) and the Japan Aerospace Exploration Agency (JAXA) Public Health Monitoring and Analysis Platform (JPMAP). Specific datasets for our analysis were used. One was the Multiple Indicator Cluster Survey (MICS), a critical survey initiative led by UNICEF and conducted across diverse countries globally [4, 5]. MICS aims to comprehensively collect data on the well-being of children and women, including their health, education, protection, and living conditions. First, the data file of the mosquito nets in households was obtained from MICS. The total sample size was 51,948 households with children under five. The questionnaires used to compile these data were collected from July to November 2017.

These MICS datasets were designed to provide estimates for a large number of indicators on the situation of children and women for three regions including North, Central and South and 18 provinces including Vientiane Capital, Phongsaly, Luangnamtha, Oudomxay, Bokeo, Luangprabang, Huaphanh, Xayabury, Xiengkhuang, Vientinae, Borikhamxay, Khammuane, Savannakhet, Saravane, Sekong, Champasack, Attapeu and Xaysomboun [5]. Within each province, the urban and rural areas were identified as the main sampling strata and the sample of 20 households was selected systematically with probability proportional to size. Five out of the 1170 selected enumeration areas were not visited because of extremely poor road conditions, households moving out, and merging of villages to neighboring villages.

The present study analyzed the household questionnaire which was used to collect basic demographic information on all household members. In the present study, to determine the factors influencing mosquito net use at bedtime in households with children under five, the following variables was collected: ethno-linguistic group of household head, education of household head, wealth index quintile, mosquito net observed, net soaked or dipped since obtained, way net was obtained, place where net was obtained, and brand/type of observed net.

The Earth observation satellite data used were obtained from the Japan Aerospace Exploration Agency (JAXA) Public Health Monitoring and Analysis Platform (JPMAP) [6]. This platform provides access to Earth observation satellite data, specifically LST records. The JPMAP utilizes MOD11/MYD11 products as LST originary provided by the National Aeronautics and Space Administration and United States Geological Survey. Also, this resource allowed us to gain insight into the environment in targeted areas worldwide. The LST is retrieved at 5 km grids by the day/night algorithm, measured four times a day, and is available from early 2000. We used average LST data for each sampling area from July to November 2017 in this research.

### Statistical analysis

The LST data from JPMAP were combined with the MICS data on household mosquito nets. Then, Chi-square tests were conducted to examine the socio-demographic characteristics of mosquito net use in Lao PDR. Finally, a bivariate logistic regression analysis was followed by a multivariate logistic regression analysis to determine the factors influencing mosquito net use at bedtime in households with children under five. Multivariate logistic regression analysis was performed using variables that were significantly related to the outcome**,** taking into account multicollinearity. Specifically, in Lao PDR, ethnicity varies depending on the area/region/province they live in. Therefore, in the final model, collinearity was taken into account and “area”, “region”, and “province” were not included. Our analysis was conducted using Stata/BE version 17.0 (StataCorp LLC, College Station, TX, USA) and SPSS version 24.0 (SPSS Inc., Chicago, IL, USA). In addition, Vientiane city, the capital of Lao PDR, was excluded from this study due to its high average temperature compared to that of other provinces and the incidence of VBDs is very low (annual parasite incidence of malaria is below 0.1 [15]).

## Results

Table 1 shows the number of household heads for each ethnicity in Lao PDR. Lao PDR is one of the most multi-ethnic countries in the world. Results indicated that the Lao-Thai lived mainly in the central area. The Mon-Khmer lived less predominantly in the center, with a prevalence in both northern and southern areas. The Hmong-Mien were not found in the south but were present from the center to the north. The Chinese-Tibetan were almost exclusively found in the north. In addition, other ethnic groups existed in central and southern areas rather than in the north.

**Table 1:** Ethnicity of heads of households in each region

Region		Lao-Tai	Mon-Khmer	Hmong-Mien	Chinese-Tibetan	Other	Total
North	n	9039	6972	2464	2038	73	20,586
	%	43.9	33.9	12.0	9.9	0.4	100
Central	n	11,390	2329	3071	5	214	17,009
	%	67.0	13.7	18.1	0.0	1.3	100
South	n	8868	5281	0	2	219	14,370
	%	61.7	36.8	0.0	0.0	1.5	100
Total	n	29,297	14,582	5535	2045	506	51,965
	%	56.4	28.1	10.7	3.9	1.0	100

As the results of the Chi-square test show in Table 2, all variables were significantly different. Among households with children under five in Lao PDR, 77.8% of the them slept under mosquito nets. Of these households, 80.5% used LLINs (Olyset, Permanent, and other brands).

**Table 2:** Socio-demographic status of the households with the households with children under five (N = 51,948)

	Persons who slept under a mosquito net last night						*p* Value^1^
	(*n* = 40,389)		(*n* = 11,559)		(*N* = 51,948)		
Land surface temperature, °C							
< 25.5	11,500	28.5	2888	25.0	14,388	27.7	< 0.001***
25.5–26.0	9369	23.2	2873	24.9	12,242	23.6	
26.0–26.5	7537	18.7	2030	17.6	9567	18.4	
26.5–27.0	2287	5.7	833	7.2	3120	6.0	
≥ 27.0	9696	24.0	2935	25.4	12,631	24.3	
Area (urban/rural with or without road)							
Urban	10,481	26.0	3702	32.0	14,183	27.3	< 0.001***
Rural with road	25,028	62.0	6691	57.9	31,719	61.1	
Rural without road	4880	12.1	1166	10.1	6046	11.6	
Area (urban/rural)							
Urban	10,481	26.0	3702	32.0	14,183	27.3	< 0.001***
Rural	29,908	74.1	7857	68.0	37,765	72.7	
Region							
North	16,514	40.9	4067	35.2	20,581	39.6	< 0.001***
Central	12,899	31.9	4105	35.5	17,004	32.7	
South	10,976	27.7	3387	29.3	14,363	27.6	
Province							
Phongsaly	1743	4.3	512	4.4	2255	4.3	< 0.001***
Luangnamtha	2146	5.3	167	1.4	2313	4.5	
Oudomxay	2493	6.2	1142	9.9	3635	7.0	
Bokeo	2029	5.0	775	6.7	2804	5.4	
Luangprabang	2701	6.7	789	6.8	3490	6.7	
Huaphanh	2701	6.7	183	1.6	2884	5.6	
Xayabury	2701	6.7	499	4.3	3200	6.2	
Xiengkhuang	2425	6.0	902	7.8	3327	6.4	
Borikhamxay	2214	5.5	823	7.1	3037	5.8	
Khammua	2622	6.5	708	6.1	3330	6.4	
Savannakhet	3425	8.5	959	8.3	4384	8.4	
Saravane	3290	8.2	729	6.3	4019	7.7	
Sekong	2418	6.0	578	5.0	2996	5.8	
Champasack	2981	7.4	1,247	10.8	4228	8.1	
Attapeu	2287	5.7	833	7.2	3120	6.0	
Xaysomboune	2213	5.5	713	6.2	2926	5.6	
Ethno-linguistic group of household head							
Lao-Tai	21,801	54.0	7485	64.8	29,286	56.4	< 0.001***
Mon-Khmer	11,951	29.6	2629	22.7	14,580	28.1	
Hmong-Mien	4537	11.2	995	8.6	5532	10.6	
Chinese-Tibetan	1670	4.1	374	3.2	2044	3.9	
Other	430	1.1	76	0.7	506	1.0	
Education of household head							
None or ECE	7529	18.7	1400	12.1	8929	17.2	< 0.001***
Primary	19,202	47.6	4968	43.0	24,170	46.5	
Lower secondary	7391	18.3	2287	19.8	9678	18.6	
Upper secondary	2067	5.1	768	6.7	2835	5.5	
Post-secondary/non-tertiary	2134	5.3	990	8.6	3124	6.0	
Higher	2025	9.8	1133	9.8	3158	6.1	
Wealth index quintile							
Poorest	8750	21.7	1588	13.7	10,338	19.9	< 0.001***
Second	10,468	25.9	2423	21.0	12,891	24.8	
Middle	9311	23.1	2827	24.5	12,138	23.4	
Fourth	7666	19.0	2699	23.4	10,365	20.0	
Richest	4194	10.4	2022	17.5	6216	12.0	
Mosquito net observed							
Observed	27,575	68.3	8327	72.0	35,902	69.1	< 0.001***
Not observed	12,814	31.7	3232	28.0	16,046	30.9	
Net soaked or dipped since obtained							
Yes	1735	11.7	252	5.9	1987	3.8	< 0.001***
No	13,015	88.0	3988	93.8	17,003	32.7	
Don’t know/not sure	35	0.2	13	0.3	48	0.1	
Way net was obtained							
Yes, ANC	565	1.4	107	0.9	672	1.3	< 0.01**
Yes, EPI	8976	22.3	2602	22.6	11,578	22.3	
No	30,749	76.3	8801	76.5	39,550	76.1	
Place where net was obtained							
Government health facility	4695	15.3	1235	14.0	5930	11.4	< 0.001***
Private health facility	53	0.2	8	0.1	61	0.1	
Pharmacy	17	0.1	3	0.0	20	0.0	
Shop/market/street	20,718	67.4	5926	67.4	26,644	51.3	
Community health worker	3485	11.3	997	11.3	4482	8.6	
Religious institution	42	0.1	10	0.1	52	0.1	
School	120	0.4	38	0.4	158	0.3	
Other	1605	5.2	579	6.6	2184	4.2	
Brand/type of observed net							
LLIN: Olyset net	6515	20.5	1330	14.1	7845	15.1	< 0.001***
LLIN: permanent net	6880	21.6	2119	22.4	8999	17.3	
LLIN: other brand	5243	16.5	1871	19.8	7114	13.7	
LLIN: don’t know brand	6965	21.9	1985	21.0	8950	17.2	
Other type	6215	19.5	2158	22.8	8373	16.1	

*ECE* early childhood education, *ANC* antenatal care, *EPI expanded programme on immunizations*, *LLIN* long-lasting insecticidal net

^1^Pearson’s Chi-square test

^*^p < 0.05, **p < 0.01, ***p < 0.001

Children under five in rural households slept under mosquito nets three times more often than did children in urban households (urban, 26.0%; rural with roads, 62.0%; and rural without roads, 12.1%).

The Lao PDR education system typically consists of four levels: primary, lower secondary, upper secondary, and tertiary education. Children are required to complete primary education in Lao PDR. However, as Table 2 reveals, around 30% of household heads had no education.

Table 2 shows that households in areas with higher LST had lower mosquito net use. Additionally, ethno-linguistic differences were observed, with Mon-Khmer and Hmong-Mien groups more likely to use mosquito nets compared to the Lao-Tai majority. Wealth index and educational level also played significant roles, as lower-income households were more likely to use mosquito nets.

Subsequently, multivariate logistic regression analysis was conducted to determine mosquito net use by children under five, with the results shown in Table 3. Four objective variables were chosen: LST, ethno-linguistic group of household head, education level of household head, and wealth index quintile, and the outcome variable was mosquito net use by households with children under five.

**Table 3:** Logistic regression analysis of determinants of mosquito net use by the households with children under five (*N* = 51,948)

	cOR (95% CI)	*p* value	aOR (95% CI)	*p* value
Land surface temperature, °C				
< 25.5	–	–	–	–
25.5–26.0	0.744	< 0.001***	0.740	< 0.001***
26.0–26.5	0.889	< 0.05*	1.083	< 0.05*
26.5–27.0	0.634	< 0.001***	0.682	< 0.001***
≥ 27.0	0.749	< 0.001***	0.848	< 0.001***
Ethno-linguistic group of household head				
Lao-Tai	–	–	–	–
Mon-Khmer	1.576	< 0.001***	1.294	< 0.001***
Hmong-Mien	1.707	< 0.001***	1.365	< 0.001***
Chinese-Tibetan	1.412	< 0.001***	0.682	0.213
Other	1.957	< 0.001***	0.848	< 0.01**
Education of household head				
None or ECE	–	–	–	–
Primary	0.708	< 0.001***	0.794	< 0.001***
Lower secondary	0.586	< 0.001***	0.708	< 0.001***
Upper secondary	0.526	< 0.001***	0.683	< 0.001***
Post-secondary/non-tertiary	0.407	< 0.001***	0.549	< 0.001***
Higher	0.336	< 0.001***	0.474	< 0.001***
Wealth index quintile				
Poorest	–	–	–	–
Second	0.757	< 0.001***	0.833	< 0.001***
Middle	0.556	< 0.001***	0.683	< 0.001***
Fourth	0.505	< 0.001***	0.680	< 0.001***
Richest	0.383	< 0.001***	0.576	< 0.001***

*cOR* crude odds ratio, *aOR* adjusted odds ratio, *CI* confidence interval, *ECE* early childhood education

^*^p < 0.05, **p < 0.01, ***p < 0.001

Mon-Khmer and Hmong-Mien were, respectively, 1.29 and 1.37 times more likely to use mosquito nets when sleeping, whereas other ethnic minorities were 0.85 times less likely to use mosquito nets compared to the Lao-Thai ethnic majority in Lao PDR. These findings suggested that differences among ethnic groups may have influenced the use of the mosquito nets in Lao PDR.

**Fig. 1:**
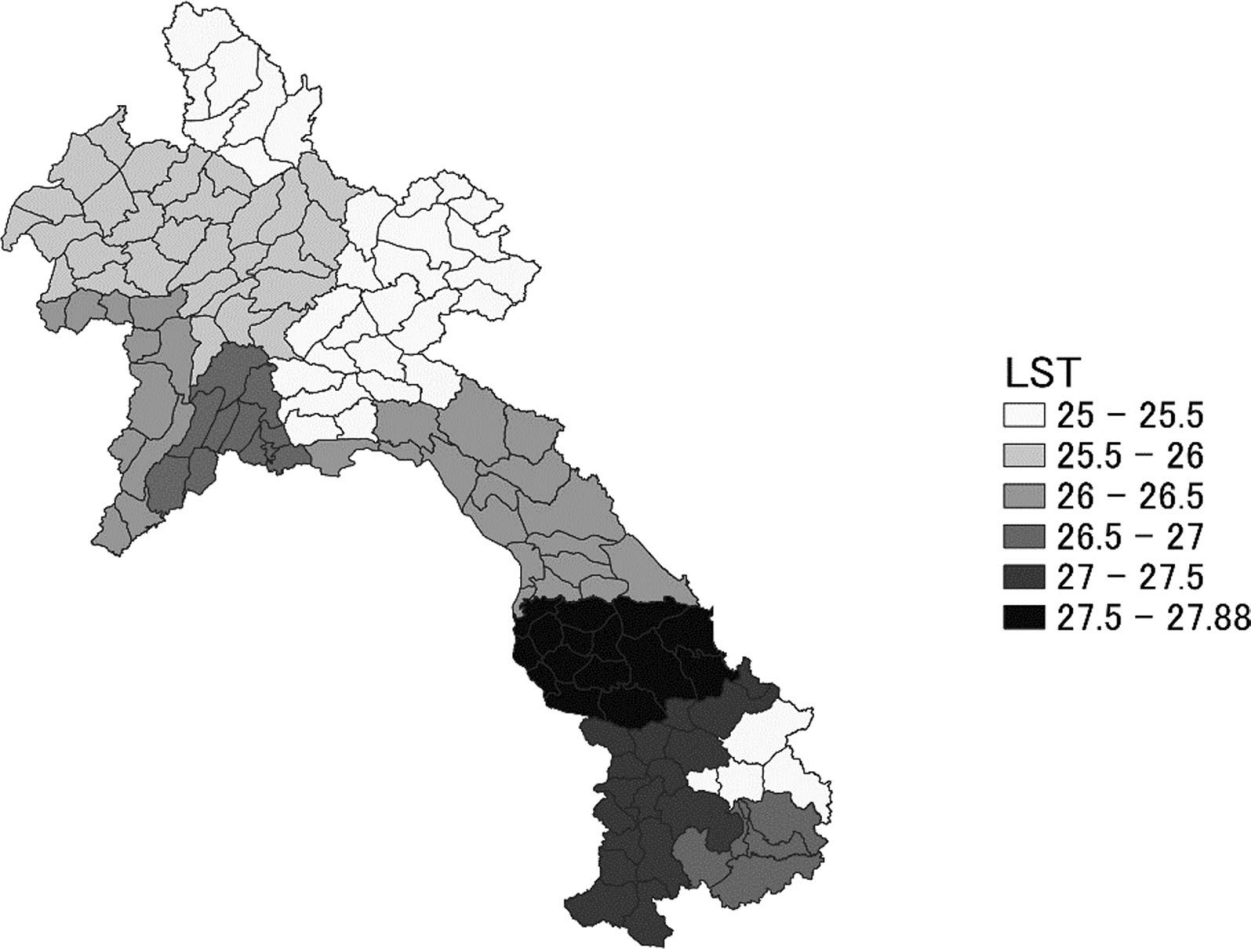
Land surface temperature (LST) map per province of Lao PDR. On this image, the temperatures are in °C

Table 3 shows that in areas with higher LST than 26.5 °C, households with children under five were less likely to use mosquito nets when sleeping. We developed the LST map of Lao PDR as shown in Fig. 1. LST was categorized into 5 categories by temperature in °C: < 25.5, 25.5–26.0, 26.0–26.5, 26.5–27.0, and 27.0 ≤ in our study. Taking into account the distribution of LST in the target area, these temperature ranges were selected due to their potential impact on mosquito behavior and human comfort. Higher temperatures can increase mosquito activity, but may also reduce the likelihood of mosquito net use due to discomfort from heat. This categorization allows us to assess how varying temperature levels influence mosquito net usage. The LST was lower in the northern and southeastern areas, which have higher elevations than in other regions, but was higher in the south and the Vientiane capital. A Chinese-Tibetan group lived in the northern area. Moreover, malaria endemic areas were mainly in the south [15], and these areas also had a higher LST.

Table 3 further indicates that the higher the education level of the household head, the fewer households use mosquito nets. Table 3 also shows that determinants of mosquito net use were associated with wealth status in Lao PDR. The use of mosquito nets tends to decrease as wealth status increases.

## Discussion

The present study aimed to assess mosquito net use and associated factors among households with children under five in Lao PDR. We found that 77.8% of all households with children under 5 years of age slept under mosquito nets. Multivariate logistic regression analysis revealed that mosquito net use was significantly associated with the LST, ethno-linguistic group, education level of the household head, and the wealth index quintile.

### Land surface temperature

Certain mean temperature ranges are favorable for some mosquitoes borne diseases transmission, whereas other mean temperature ranges are unfavorable. Moreover, even with the same mean temperature higher diurnal temperature ranges are unfavorable for mosquitoes borne diseases transmission than lower diurnal temperature ranges [16]. The effectiveness of mosquito net use is closely linked to these temperature variations. The analysis of mosquito net use in Lao PDR reveals significant insights into the impact of LST on malaria prevention. Higher temperatures play an important role in mosquito development and behavior and influence the effectiveness of malaria control measures [17]. The present study found that higher LST, particularly more than 26.5 °C, correlated with increased mosquito net use (*p* < 0.001). This suggests that higher temperatures may deter individuals from using mosquito nets due to discomfort or perceptions of lower mosquito activity [18, 19]. The belief that mosquito nets cause discomfort due to heat may be exaggerated; while they can reduce airflow and slightly increase temperature, this increase is often minimal [20]. Moreover, slight discomfort should not deter individuals from using mosquito nets as the health benefits of preventing mosquito-borne diseases far outweigh the minor inconvenience. Health authorities should educate the public that while the use of ITNs might cause a slight increase in temperature due to reduced airflow, this discomfort is minor. The protective health benefits against mosquito-borne diseases, particularly malaria, far outweigh the slight increase in warmth. It is important to communicate these benefits effectively, as consistent net usage can significantly reduce malaria transmission and improve public health outcomes.

### Education level

The relationship between education level and mosquito net use in Lao PDR is complex and significant. Findings from the Chi-square test indicated that around 17.2% of household heads in Lao PDR had no education, and these households were more likely to use mosquito nets. This regression suggests that lower educational attainment is linked to higher reliance on mosquito nets. Households with higher education levels were less likely to use mosquito nets. Hence, the education level of household heads might also be associated with mosquito net use in Lao PDR.

The logistic regression analysis further highlighted the significance of the education level of household heads as a determinant of mosquito net use. Malaria prevention measures using mosquito nets are still needed for low-education households, because there are still some households not using them. Mosquito nets are a low-cost and effective method [1], and malaria prevention measures need to be thoroughly implemented through policies, especially for households with low levels of education.

### Ethnic groups

The study also revealed disparities based on ethnic and socio-demographic factors. There was a notable regression between the region and the ethnic-linguistic group of household heads. Different ethnic groups show varying likelihoods of using mosquito nets. Compared to the Lao-Thai ethnic majority in Lao PDR, Mon-Khmer and Mon-Mien groups were, respectively, 1.29 and 1.37 times more likely to use mosquito nets when sleeping, whereas other ethnic minorities were 0.85 times less likely to use mosquito nets. These findings suggested that differences among ethnic groups may have influenced the use of the mosquito nets in Lao PDR.

Disparities in mosquito net use between urban and rural households were also found, with rural households more often using nets. The usage rates were 26.0% in urban areas, 62.0% in rural areas with roads, and only 12.1% in rural areas without roads. These results showing a huge difference in rural areas with and without roads indicated that mosquito nets might not reach areas with poor. This disparity underscores the need for infrastructure improvements to enhance access to malaria prevention tools in remote areas.

### Wealth status

The present study further revealed a relationship between wealth status and the use of mosquito nets. Wealthier households tended to use mosquito nets less frequently than poorer households. This trend can be attributed to several factors, including differences in housing quality, access to alternative preventive measures, and overall living conditions. For example, whether the walls are made of plants with gaps or concrete makes a difference in the ease of entry of vector mosquitoes. Poorer households tend to adopt more traditional forms of housing using straw or palm leaves, which require more mosquito nets. Wealthier households, however, tend to live in relatively more secure houses, complete with concrete walls, air conditioning, and other amenities. The ability to purchase oral prophylaxis for malaria also depends on whether one has the financial means to do so.

This finding supports previous research suggesting a link between malaria prevention and its incidence. Members of poorer socio-economic groups often live in dwellings that offer minimal protection against mosquitoes and cannot afford ITNs [21]. In poorer households, the use of mosquito nets is more prevalent due to higher susceptibility to mosquito infiltration. These households often have less secure housing structures, such as those made from traditional materials like straw or palm leaves, which provide fewer barriers against mosquitoes. Consequently, mosquito nets become a crucial preventive measure to reduce malaria transmission [22]. In addition, wealthier households have better housing conditions, including more secure, modern structures that reduce mosquito entry, and access to air conditioning, which can lower mosquito activity indoors. As a result, the reliance on mosquito nets decreases [23].

## Limitation

The strength of this study is that it provides specific evidence for measures against mosquito-borne infectious diseases in a country with limited resources, using a detailed epidemiological data set covering the entire Lao PDR and a detailed data set from a high-performance Earth observation satellite. On the other hand, the limitations of this study include the lack of a detailed ethnic setting or mosquitoes (species, etc.) data, and we expect to conduct more detailed analyses in the future.

## Conclusion

To bring Lao PDR closer to the day when mosquito-borne infectious disease like malaria are eliminated, the analysis of the present study suggest measures to intensify the use of mosquito nets, with emphasis on ethnic minorities (except Lao-Tai, Mon-Khmer, Hmong-Mienm, and Chinese-Tibetan) living in hot areas.

## Supplementary Information

Additional file 1.

## Data Availability

Data and materials have been provided in the main manuscript.

## Abbreviations

ITN: Insecticide-treated nets
JAXA: Japan Aerospace Exploration Agency
JPMAP: JAXA’s Public Health Monitoring and Analysis Platform
Lao PDR: Lao People’s Democratic Republic
LLINs: Long-lasting insecticidal nets
LST: Land surface temperature
MICS: Multiple Indicator Cluster Survey
WHO: World Health Organization

## References

[1] WHO, World Malaria Report. 2023. https://www.who.int/teams/globalmalaria-programme/reports/world-malariareport-2023. Accessed 26 Nov 2024.

[2] Fischer L, Gültekin N, Kaelin MB, Fehr J, Schlagenhauf P. Rising temperature and its impact on receptivity to malaria transmission in Europe: a systematic review. Travel Med Infect Dis. 2020;36: 101815. 32629138 10.1016/j.tmaid.2020.101815

[3] Hill J, Lines J, Rowland M. Insecticide-treated nets. Adv Parasitol. 2006;61:77–128. 16735163 10.1016/S0065-308X(05)61003-2

[4] WHO. Insecticide-treated mosquito Nets: a WHO Position Statement. 2007. http://www.ivcc.com/sites/ivcc.mrmdev.co.uk/files/content/itnspospaperfinal.pdf. Accessed 19 Oct 2024.

[5] The DHS Program. Lao PDR Lao Social Indicator Survey II (LSIS II), Lao PDR. 2018. https://dhsprogram.com/pubs/pdf/FR356/FR356.pdf. Accessed 19 Oct 2024.

[6] Zhou Y, Zhang WX, Tembo E, Xie MZ, Zhang SS, Wang XR, et al. Effectiveness of indoor residual spraying on malaria control: a systematic review and meta-analysis. Infect Dis Poverty. 2022;11(1):83. 35870946 10.1186/s40249-022-01005-8 PMC9308352

[7] Vilay P, Nonaka D, Senamonty P, Lao M, Iwagami M, Kobayashi J, et al. Malaria prevalence, knowledge, perception, preventive and treatment behavior among military in Champasak and Attapeu provinces, Lao PDR: a mixed methods study. Trop Med Health. 2019;47:11. 30700970 10.1186/s41182-019-0138-9 PMC6347756

[8] Lao PDR Peace Independence Democracy Unity Prosperity. Malaria NSP 2021–2025 Lao PDR. 2020. .https://apmen.org/sites/default/files/all_resources/Lao%20PDR%20National%20Strategic%20Plan%20for%20Malaria%202021-2025.pdf#. Accessed 19 Oct 2024.

[9] Zafar S, Overgaard H, Pongvongsa T, Vannavong N, Phommachanh S, Shipin O, et al. Epidemiological profile of dengue in Champasak and Savannakhet provinces, Lao People’s Democratic Republic, 2003–2020. Western Pac Surveill Response J. 2022;13(4):30–42. 10.5365/wpsar.2022.13.4.932 PMC9912291 36817500

[10] Phanhkongsy S, Suwannatrai A, Thinkhamrop K, Somlor S, Sorsavanh T, Tavinyan V, et al. Spatial analysis of dengue fever incidence and serotype distribution in Vientiane Capital, Laos: a multi-year study. Acta Trop. 2024;256: 107229. 38768698 10.1016/j.actatropica.2024.107229

[11] Senavong P, Yamamoto E, Keomoungkhoune P, Prasith N, Somoulay V, Kariya T, et al. Factors associated with severe dengue in Savannakhet Province, Lao People’s Democratic Republic. Nagoya J Med Sci. 2021;83(4):749–63. 34916719 10.18999/nagjms.83.4.749 PMC8648533

[12] Winskill P, Walker PG, Cibulskis RE, Ghani AC. Prioritizing the scale-up of interventions for malaria control and elimination. Malar J. 2019;18(1):122. 30961603 10.1186/s12936-019-2755-5 PMC6454681

[13] Kleinschmidt I, Bradley J, Knox TB, Mnzava AP, Kafy HT, Mbogo C, et al. Implications of insecticide resistance for malaria vector control with long-lasting insecticidal nets: a WHO-coordinated, prospective, international, observational cohort study. Lancet Infect Dis. 2018;18(6):640–9. 29650424 10.1016/S1473-3099(18)30172-5 PMC5968369

[14] Rotejanaprasert C, Malaphone V, Mayxay M, Chindavongsa K, Banouvong V, Khamlome B, et al. Malaria epidemiology, surveillance and response for elimination in Lao PDR. Infect Dis Poverty. 2024;13(1):35. 38783374 10.1186/s40249-024-01202-7 PMC11112833

[15] Matsumoto-Takahashi ELA, Iwagami M, Oyoshi K, Sasaki Y, Hongvanthong B, Kano S. Deforestation inhibits malaria transmission in Lao PDR: a spatial epidemiology using Earth observation satellites. Trop Med Health. 2023;51(1):60. 37915065 10.1186/s41182-023-00554-4 PMC10621094

[16] Talesnik D. NIH record. Researchers discuss impact of climate change on mosquito-borne diseases. https://nihrecord.nih.gov/2022/09/16/researchers-discuss-impact-climate-change-mosquito-borne-diseases. Accessed 1 Aug 2024.

[17] Pourtois JD, Tallam K, Jones I, Hyde E, Chamberlin AJ, Evans MV, et al. Climatic, land-use and socio-economic factors can predict malaria dynamics at fine spatial scales relevant to local health actors: evidence from rural Madagascar. PLOS Glob Public Health. 2023;3(2): e0001607. 36963091 10.1371/journal.pgph.0001607 PMC10021226

[18] Shapiro LLM, Whitehead SA, Thomas MB. Quantifying the effects of temperature on mosquito and parasite traits that determine the transmission potential of human malaria. PLoS Biol. 2017;15(10): e2003489. 29036170 10.1371/journal.pbio.2003489 PMC5658182

[19] Ricotta E, Oppong S, Yukich JO, Briët OJT. Determinants of bed net use conditional on access in population surveys in Ghana. Malar J. 2019;18(1):63. 30849976 10.1186/s12936-019-2700-7 PMC6408824

[20] Ndjinga JK, Minakawa N. The importance of education to increase the use of bed nets in villages outside of Kinshasa, Democratic Republic of the Congo. Malar J. 2010;9(1):279. 20937157 10.1186/1475-2875-9-279 PMC2959078

[21] Onwujekwe O, Uzochukwu B, Dike N, Okoli C, Eze S, Chukwuogo O. Are there geographic and socio-economic differences in incidence, burden and prevention of malaria? A study in southeast Nigeria. Int J Equity Health. 2009;8(1):45. 20030827 10.1186/1475-9276-8-45 PMC2806339

[22] Noor AM, Kinyoki DK, Mundia CW, Kabaria CW, Mutua JW, Alegana VA, et al. The changing risk of *Plasmodium falciparum* malaria infection in Africa: 2000–10: a spatial and temporal analysis of transmission intensity. Lancet. 2014;383(9930):1739–47. 24559537 10.1016/S0140-6736(13)62566-0 PMC4030588

[23] Sewe MO, Ahlm C, Rocklöv J. Remotely sensed environmental conditions and malaria mortality in three malaria endemic regions in Western Kenya. PLoS ONE. 2016;11(4): e0154204. 27115874 10.1371/journal.pone.0154204 PMC4845989

