# Supplementary material for: Higher temperatures and being an ethnic minority reduce mosquito net use in Lao PDR: an analysis of Lao PDR’s Multiple Indicator Cluster Survey and Earth observation satellite data
Source: Trop Med Health. 2024 Dec 24;52:99. doi: 10.1186/s41182-024-00669-2 (PMC11667946; doi:10.1186/s41182-024-00669-2)
Supplement: Supplementary file 1 — Additional file 1. [file 41182_2024_669_MOESM1_ESM.docx]

**Variable Definitions**

The following variables were analyzed in this study, with detailed descriptions provided below:

The temperature of the Earth's surface as measured by remote sensing methods is known as the "land surface temperature" (LST). The Japan Aerospace Exploration Agency (JAXA) Public Health Monitoring and Analysis Platform (JPMAP) provided the LST data used in this study, which were measured in degrees Celsius. Five temperature ranges were identified by averaging the LST values from July to November 2017: <25.5°C, 25.5-26.0°C, 26.0-26.5°C, 26.5-27.0°C, and ≥27.0°C. This variable is crucial as temperature impacts mosquito behavior, breeding cycles, and malaria transmission.

Ethno-linguistic Group of Household Head: The ethno-linguistic group of the household head was self-reported during the MICS survey. The major groups represented in this study include Lao-Tai, Mon-Khmer, Hmong-Mien, Chinese-Tibetan, and Other. Understanding ethno-linguistic differences is important for identifying variations in health behaviours, including the use of mosquito nets.

Education Level of Household Head: The education level of the household head was categorized into six levels: No formal education or Early Childhood Education (ECE), Primary education, Lower secondary education, Upper secondary education, Post-secondary/non-tertiary education, and Higher education. Education level is an important determinant of health literacy and access to healthcare services, which may influence mosquito net usage.

Wealth Index Quintile: The wealth index was used to measure the household’s standard of living and was constructed based on asset ownership, housing characteristics, and access to services. The index divided households into five quintiles: Poorest, Second, Middle, Fourth, and Richest. Wealth status can affect access to mosquito nets and the type of housing that provides protection against mosquitoes.
